# Human-murine chimeric autoantibodies with high affinity and specificity for systemic sclerosis

**DOI:** 10.3389/fimmu.2023.1127849

**Published:** 2023-06-16

**Authors:** Sunhui Chen, Qiong Liang, Yanhang Zhuo, Qin Hong

**Affiliations:** ^1^ Shengli Clinical Medical College of Fujian Medical University, Fujian Provincial Hospital, Fuzhou, China; ^2^ Department of Pharmacy, Fujian Provincial Hospital, Fuzhou, China; ^3^ Center for Experimental Research in Clinical Medicine, Fujian Provincial Hospital, Fuzhou, China; ^4^ Fujian Provincial Key Laboratory of Critical Care Medicine, Fujian Provincial Hospital, Fuzhou, China

**Keywords:** systemic sclerosis, ATA, phage display, humanized antibody, antibody modeling

## Abstract

Scleroderma 70 (Scl-70) is commonly used in the clinic for aiding systemic sclerosis (SSc) diagnosis due to its recognition as autoantibodies in the serum of SSc patients. However, obtaining sera positive for anti-Scl-70 antibody can be challenging; therefore, there is an urgent need to develop a specific, sensitive, and easily available reference for SSc diagnosis. In this study, murine-sourced scFv library were screened by phage display technology against human Scl-70, and the scFvs with high affinity were constructed into humanized antibodies for clinical application. Finally, ten high-affinity scFv fragments were obtained. Three fragments (2A, 2AB, and 2HD) were select for humanization. The physicochemical properties of the amino acid sequence, three-dimensional structural basis, and electrostatic potential distribution of the protein surface of different scFv fragments revealed differences in the electrostatic potential of their CDR regions determined their affinity for Scl-70 and expression. Notably, the specificity test showed the half-maximal effective concentration values of the three humanized antibodies were lower than that of positive patient serum. Moreover, these humanized antibodies showed high specificity for Scl-70 in diagnostic immunoassays for ANA. Among these three antibodies, 2A exhibited most positive electrostatic potential on the surface of the CDRs and highest affinity and specificity for Scl-70 but with least expression level; thus, it may provide new foundations for developing enhanced diagnostic strategies for SSc.

## Introduction

Systemic sclerosis (SSc) is a rare, chronic, autoimmune rheumatic disease characterized by excessive inflammation and collagen affecting several organs (1). Moreover, its progression is usually accompanied by the production of autoantibodies against major antigens. Anti-centromere antibodies, anti-topoisomerase I antibodies (ATA), and anti-RNA-polymerase III antibodies were three most important diagnosis and prognostic marker for SSc (2). ATA, formerly anti-Scl70 antibodies, are present in 3–75% of patients with SSc, with a sensitivity of 34%, which increases to 40% in patients with diffuse cutaneous SSc (3, 4). Thus, ATA are the primary diagnostic marker for SSc; additionally, anti-Scl-70 positivity can predict people with high risk of interstitial fibrosis/restrictive lung disease (5).

Four methods are used for the detection of ATA: immunodiffusion, western blotting, immunoprecipitation, and enzyme-linked immunosorbent assay (ELISA). Among these, immunodiffusion is the gold standard, whereas ELISA is highly time-efficient and inexpensive. Most of the positive controls in commercial ELISA kits for SSc diagnosis originate from human serum or plasma. However, with increasing attention on biosecurity and ethical supervision of human genetics, limited sources and utility of human serum or plasma containing known specific antibodies have become a challenge for the development and production of autoimmune disease reagents (6). Therefore, it is valuable to develop an efficient and convenient strategy to generate antibody substituted for SSc patient serum. The phage display technology is a powerful tool for the generation of high affinity single chain variable fragment antibodies (scFv) (7). This technique is particularly advantageous because it allows for the selection of scFv against a wide range of targets, including membrane proteins and molecules that are difficult to express or purify. Moreover, the phage display method is highly sensitive, enabling the detection of low-abundance antigens (7). In 2003, Weber et al. isolated B cells from a small amount of patient blood to generate the scFvs libraries by the phage display technology, and obtained the four anti-Scl-70 scFvs through phage screening (8). In contrast with these demerits on the use of native human antibodies, humanized monoclonal antibodies (mAbs) can reduce heterologous interference of mouse antibodies and also have several advantages, such as high specificity and affinity, small inter-batch difference, and easy industrial mass production (9). Humanized mAb improves the structure of traditional antibodies, making their application more targeted, functional, and operational, with the ability to achieve many functions that traditional antibodies cannot (10). However, a humanized anti-Scl-70 antibody substitute for positive patient serum has not been reported yet.

In this study, three anti-Scl-70 humanized antibodies from murine were characterized as specific, sensitive, and easily available artificial substitute for positive reference of SSc diagnosis. Physicochemical properties, three-dimensional structural basis, and electrostatic potential distribution of the protein surface of scFv were evaluated. The findings of this study are expected to provide scientific basis for enhanced SSc diagnostic method development.

## Materials and methods

### Immunization of mice with recombinant human Scl-70

Three female 6–8 weeks old Balb/c mice (Shanghai SLAC Laboratory Animal Co., Shanghai, China), were injected subcutaneously with 100 µg recombinant human Scl-70 protein expressed by recombinant baculovirus infection of *Spodoptera frugiperda* Sf9 insect cells (Surmodics IVD, Eden Prairie, MN, USA). At 2-week intervals, four boosters of 50 µg recombinant human Scl-70 emulsified in an equal amount of incomplete Freund’s adjuvant (Sigma-Aldrich, St. Louis, MO, USA) were administered. Seven days after the last booster inoculation with 100 µg of Scl-70, the mice were euthanized using 100% CO_2_ and their spleens were collected and stored at −80 °C. The animal experiments were approved by the Institutional Animal Care and Use Committee of Fudan University (No. 8177141042).

### Construction of mice scFv library

Total RNA was prepared using Invitrogen TRIzol Reagent (Thermo Fisher Scientific, Waltham, MA, USA) and cDNA was amplified using Prime Script RT Master Mix (Takara Bio, Kusatsu, China). Primers used for the amplification of the mouse VL and VH genes were previously described (11) (**Table S1**). A mixture (50 µL) containing 10 nmol of each forward and reverse primer, 4 µL cDNA extracted from spleen tissue of the immunized mice, 0.25 mmol dNTP mixture (Takara Bio), 10× buffer, and 0.5 U ExTaq DNA polymerase (Takara Bio) was prepared. The amplification conditions were as follows: initial denaturation at 95 °C for 5 min; 35 cycles of 95 °C for 60 s, 55 °C for 60 s, and 68 °C for 60 s; and a final extension step at 72 °C for 10 min. The amplified products of the mouse VL and VH genes were purified using a QIAquick Gel Extraction Kit (Qiagen, Hilden, Germany).

Splicing overlap extension polymerase chain reaction (SOE-PCR) was performed to generate the mouse scFv gene. Briefly, the first-step PCR products of heavy and light chains were mixed and the assembly PCR containing equal amounts of the products was cycled 20 times at 96 °C for 60 s, 63 °C for 60 s, and 68 °C for 60 s without primers. The second PCR products (scFv gene) were amplified using the Y15 and Y16 primers. The third overlap PCR was performed under the same cycling conditions as the first PCR at an annealing temperature of 65 °C. ScFv was cloned into the phage plasmid pCANTAB5E digested with *Not*I and *Sfi*I using T4 DNA ligase (Novagen, San Diego, CA, USA) at 16 °C for 12 h. The recombinant plasmid pCANTAB5E-scFv was introduced into SS320 cells using a Gene Pulser (Bio-Rad Laboratories, Hercules, CA, USA). For phage particle packaging, the transformed SS320 cells were infected with the M13K07 helper phage (stock concentration: 10^11^ pfu/mL) according to a previously described protocol (12).

### Diversity analysis of the antibody library

Primary phage particles (1 µL) were diluted (1:10, 1:10^3^, and 1:10^5^) and then 10 µL was used to infect 100 μL of SS320 cells for 30 min at 37 °C without shaking. Then, 10 μL droplets of each dilution series were spotted on single 2YT-tetracycline and 2YT-kanamycin agar plates and incubated at 37 °C for 12 h. The number of colonies was calculated based on the capacity of the constructed phage library. Single colonies were selected and cultured overnight, and the plasmids were extracted. Plasmids were sent to Sangon Biotech Co. (Shanghai, China) for sequencing. The diversity of the library was assessed based on sequence analysis of the framework region (FR) and complementarity determining region (CDR) from the IMGT database (13).

### Selection of anti-Scl-70 scFvs by panning with magnetic beads and immunotubes

Four rounds of panning were performed for affinity phage selection. Panning was performed for the first and third rounds based on magnetic beads with 100 μg/mL and 30 μg/mL biotin-labeled Scl-70, respectively, while the second and final rounds were performed in tubes coated with 100 and 30 nM recombinant Scl-70, respectively. The stringency of the selection conditions was increased through four subsequent rounds of panning. The Scl-70 antigen concentration decreased and wash times increased (1, 5, 15, and 30 min) with each subsequent round.

Magnetic bead-based phage display panning was performed as described previously (14, 15). Briefly, 1×10^11^ phage particles were preincubated in 100 μL of 2.5% BSA and 20 μL of unconjugated streptavidin magnetic beads (Invitrogen) for 1–2 h at room temperature on a rotator. The unconjugated streptavidin magnetic beads were then removed. The solution containing the remaining phages was collected and incubated with 1.5 μg biotinylated Scl-70 for 1 h. Then, 1 mg pre-blocked magnetic streptavidin beads was added and the mixture was rotated for 15 min. Beads were magnetically harvested from the solution, washed several times with phosphate-buffered saline (PBS) containing 0.05% Tween-20 (PBST), and twice with PBS. Bound phages were eluted by incubating the washed beads with 1 mL trypsin (10 g/L in PBS, pH 7.4, and pre-warmed at 37 °C) for 30 min at room temperature. Beads were harvested and discarded, and the solution containing the eluted phages was transferred to a clean tube and incubated with 1 mL of 5% newborn serum (Invitrogen) for 5 min to inhibit trypsin activity. Phages were amplified using a trypsin-sensitive helper phage and purified from the medium using polyethylene glycol precipitation (16). Regular phage library selection on the immunotube was performed as previously described (14, 15).

### Phage titration

The titers of the phage input and output were calculated as previously described (12). The serially diluted phages were used to infect 100 μL electrocompetent cells (SS320) for 30 min at 37 °C without shaking. Then, 10 μL droplets of each dilution series were spotted on single 2YT-tetracycline and 2YT-kanamycin agar plates to check the rescue and enrichment of the antibody phage. Finally, the plates were incubated top-down/on at 37 °C after drying the droplets. The whole process was performed using filter tips to prevent contamination of the SS320 cells and pipette.

### Phage ELISA

The efficiency of every round of panning was evaluated based on the specificity of the output phages to Scl-70, which was analyzed using polyclonal phage ELISA. Briefly, 30 µL of Scl-70 (2 µg/mL) was coated onto separate wells of a 96-well MaxiSorp plate overnight at 4 °C. After blocking with PBS adding 5% milk (PBSM), a 1:10 dilution of phages obtained from each round of panning was added to the wells and incubated for 1 h at room temperature. After washing with PBST, 30 µL horseradish peroxidase (HRP)-conjugated anti-M13 antibody (Amersham Pharmacia Biotech, Amersham, UK. 1:2,0000 dilution in 5% PBSM) was added to the wells and incubated for 1 h at room temperature. The plate was finally washed with PBS, and the reactions were developed using tetramethylbenzidine substrate (Amresco, Solon, OH, USA). Helper phages were used as negative controls.

For single phage ELISA, individual clones were picked for every round of biopanning, grown at 37 °C in 96-well plates, and rescued with M13KO7 helper phage, as described previously (12). Their reactivity with Scl-70 was analyzed using phage ELISA as described above. A sample optical density at 450 nm (OD_450_) value/negative control OD_450_ value (S/N ratio) > 2 was determined as the positive standard. The sample with an OD_450_ value in the PBS-coated plate > OD_450_ value in Scl-70 coated plate was determined to be a non-specific binding clone. Phages specific for other antigens were used as negative controls.

### Expression and purification of murine-human chimeric Scl-70-Fab

The sequences of the selected colonies were inserted into a pcDNA 3.4-TOPO vector with Fc fragment of human IgG1 which constructed by Sino Biological lnc. as described in protocol (17) for mammalian cell expression using the Gibco ExpiCHO Expression System Kit (Thermo Fisher Scientific). After 7 days of cell culture at 32 °C, recombinant proteins were purified from cell culture supernatants by affinity chromatography of protein A and size-exclusion gel chromatography (SEC) using a XBridge BEH200Å column (3.5 μm, 7.8×150 mm; Waters, Milford, MA, USA) packed in a high-performance liquid chromatography (HPLC) system (Agilent 1260; Agilent Technologies, Santa Clara, CA, USA). The mobile phase was aqueous (0.15 M PBS, pH 7.4) with a 0.8 mL/min flow rate. The sample injection volume was 20 μL. The column temperature was set to 30 °C. The ultraviolet detector was set at 280 nm, with a reference wavelength of 360 nm. The expression of recombinant proteins was confirmed by sodium dodecyl-sulfate polyacrylamide gel electrophoresis and Coomassie blue staining.

### Determination of IgG affinity by ELISA

ELISA plate wells were coated with 2 µg/mL Scl-70 at 4 °C overnight. After blocking with PBS containing 5% milk at room temperature for 2 h, serially diluted phages and antibodies were incubated at room temperature for 1 h. The anti-human-IgG-Fc-HRP (Invitrogen A18817) was diluted to 1:5,000 as secondary antibody. The enzyme reaction was then performed using tetramethylbenzidine as substrate, and color development was stopped by adding 2 M H_2_SO_4_. The absorbance at 450 nm was measured using a microplate reader (18). For antibody affinity, ipilimumab was used as negative control.

### Determining specificity of murine-human chimeric antibodies

ANA pro ELISA assay Kit (ANP31-K01; Eagle Biosciences, Amherst, NH, USA) was used to determine the specificity of the murine-human chimeric antibodies. Positive patient serum was collected from patients with SSc upon approval from the Ethics Committee of Fujian Provincial Hospital, Fuzhou, China (No. K2022-04-010).

### Antigen and antibody modeling

First, the protein structure of the antigen Scl-70 was obtained from the RCSB Protein Data Bank (https://www.rcsb.org; PDBID: 1r49). The sequences of the four antigen epitopes recognized by positive serum from patients (19, 20) were labeled using PyMOL (21). Three-dimensional models of antibodies were generated using antibody homology modeling protocols from Rosetta Antibody (22, 23). Briefly, the sequence of the antibody split into components was explored for the closest structural segment using a BLAST-based method, and the segments were then assembled into a template model. After the initial V_L–_V_H_ orientation and grafting CDR templates were established, the H3 loop was completely remodeled, refined, repacked, and minimized using the next-generation KIC loop modeling protocol (24) and a rigid-backbone Rosetta Dock protocol (24, 25). The models were evaluated using the Rosetta score. Finally, the lowest-scoring model was visualized using PyMOL.

### Statistical analysis

All data were analyzed using Prism 5 (GraphPad Software, San Diego, CA, USA). Comparisons between two groups were conducted using two-tailed Student’s *t*-test, and analysis of variance with Tukey’s *post-hoc* tests were performed for comparisons among multiple groups. The statistical significance was set at *p* < 0.05.

## Results

### Animal immunization

**Figure 1:**
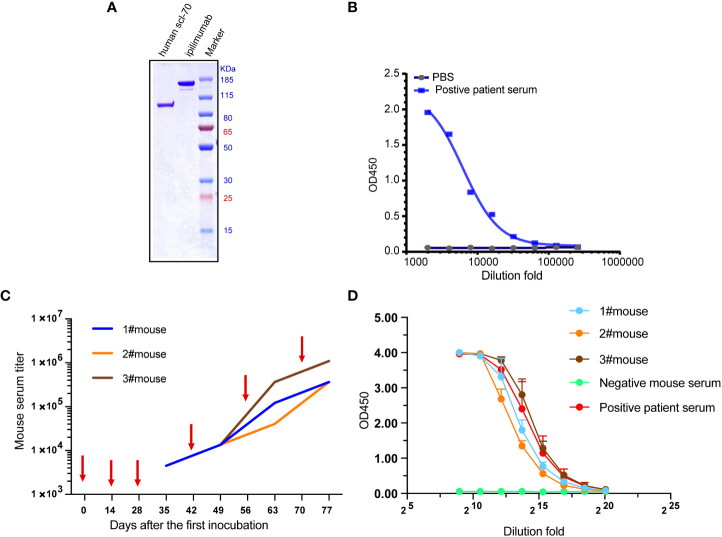
Immunization of three Balb/c mice with recombinant human Scl-70 antigen. **(A)** SDS-PAGE showing the molecular weight of the Scl-70 antigen was approximately 91 kDa. **(B)** Recombinant human Scl-70 antigen react against the positive patient serum. **(C)** Titers of immunized mice serum against Scl-70 antigen increased following each immunization (depicted with arrows). **(D)** The serum from the immunized mice after incubation of the last booster react with antigen comparable to the positive patient serum.

The result of non-reducing SDS-PAGE showed the apparent molecular mass (Mr) of recombinant Scl-70 matches the pronounced Mr of 91 kDa (**Figure 1A**), and ELISA result showed the recombinant Scl-70 antigen react against the positive patient serum directly (**Figure 1B**). The animals were then immunized six times with recombinant human Scl-70, following the immunization schedule, and serum was collected 7 days after every incubation. Serum titers of mice rose gradually after every immunization (**Figure 1C**). Finally, after the last booster, mice sera reacted against Scl-70 comparable to positive patient serum after 1×10^5^–1×10^6^ -fold dilution (**Figure 1D**).

### Construction of recombinant phage library

Total RNA extracted from spleen cells of immunized mice was reverse-transcribed into cDNA. The procedure used to construct the recombinant phage library and immunized Fab library as showed in **Figure S1**, which contained 3.4×10^8^ independent clones. Seventy-two positive clones were randomly selected from the library for sequencing. Compared with the IMGT database, the results indicated that the alignment and length of the insertion sequence were correct, which matched the basic structure of the mouse antibody. Unique sequences accounted for 100% of the results and 84.7% of the colonies were correctly expressed. Overall, the mouse Scl-70-Fab phage display library showed high diversity. Then, the bacterial transformants were infected with helper phages to rescue phagemid particles displaying Fab on the phage tip.

### Panning of scFv clones against Scl-70

**Figure 2:**
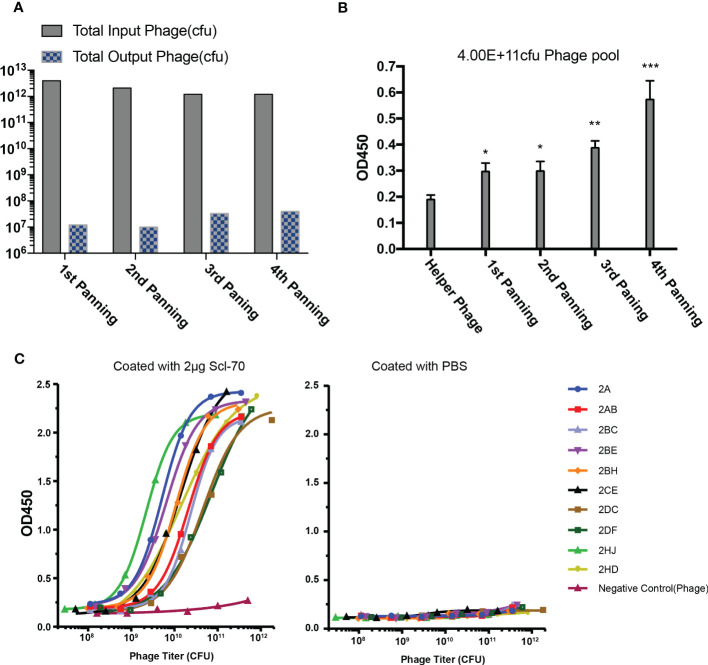
Biopanning of recombinant phage library for human Scl-70 specific scFv. **(A)** Input and output titers of recombinant phages after four rounds of panning. **(B)** Affinity of the pooled phages climbed after each panning round determined by polyclonal phage ELISA. Data represents the mean ± standard deviation of one experiment performed with duplicates. Significance was determined by two-tailed unpaired Student’s *t*-tests. *p < 0.05; **p < 0.01; ***p < 0.001. **(C)** Affinity determination of the positive scFv clones determined by monoclonal phage ELISA.

**Figure 3:**
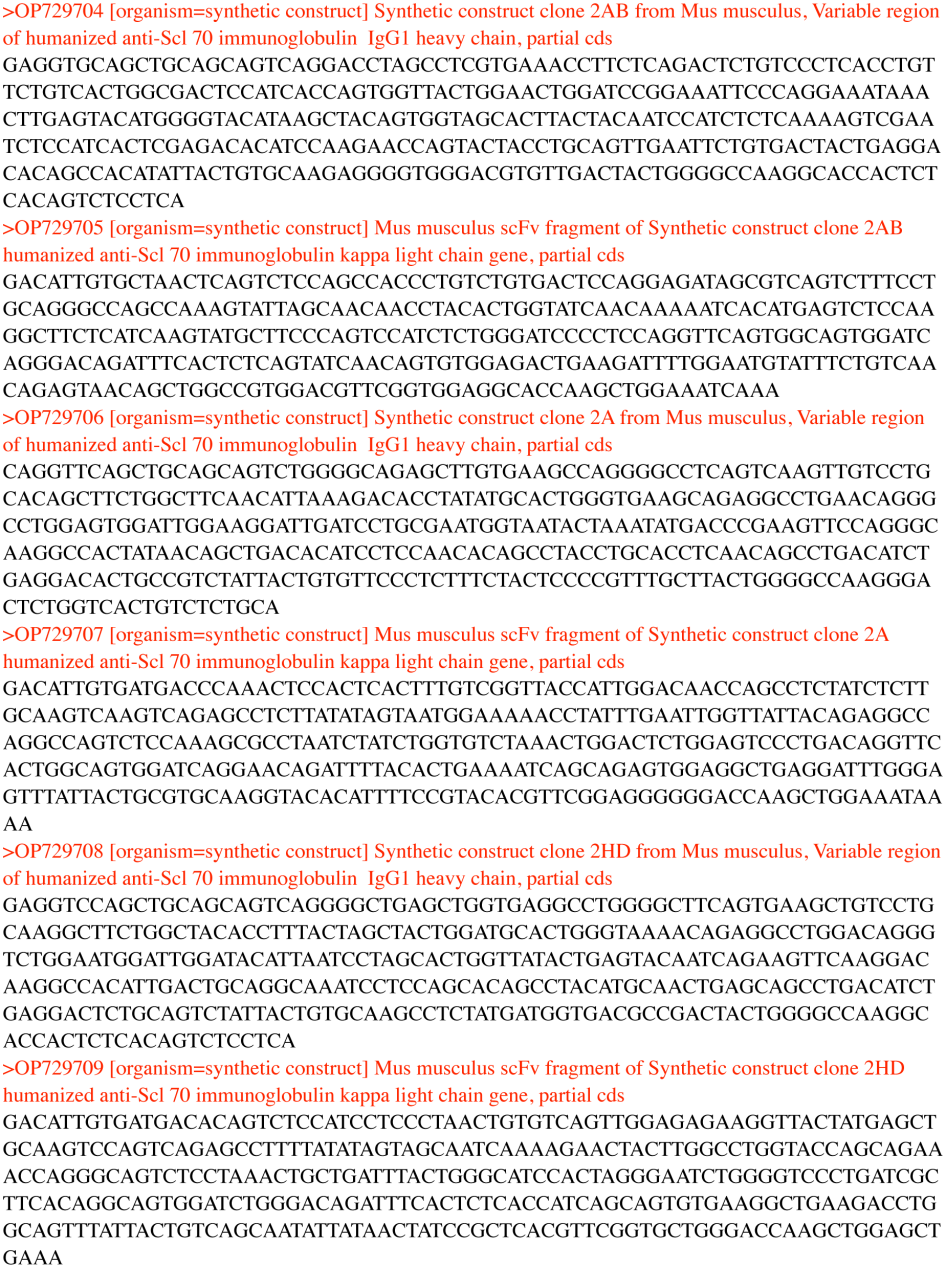
The sequences of the genes encoding the three antibodies 2A, 2AB and 2HD and the accession numbers.

Phage libraries were panned with streptavidin magnetic beads and immunotubes at staggered times for four rounds, with the titer of the eluted phages after each panning round increasing distinctly and maintaining a stable level of approximately 10^7^ pfu/mL (**Figure 2A**), which indicated that more specific phages were enriched in the process of biopanning. The output phages from every biopanning round demonstrated increased affinity for the Scl-70 antigen (**Figure 2B**), as determined by polyclonal phage ELISA. After four rounds of biopanning, 808 colonies were amplified and examined by single-colony phage ELISA, of which 168 positive phage single colonies were identified for sequencing. The affinity of 47 phage colonies with unique sequences was determined by ELISA, and 10 phages exhibited strong binding activity to the Scl-70 antigen (**Figure 2C**). Among these phages, three colonies (2A, 2AB, and 2HD) were selected for humanization (**Table 1**). The sequences of them and the accession numbers were provided in **Figure 3**.

**Table 1:** Germline information of the three Fabs with unique sequence.

Clone ID	Light chain *V*	Heavy chain *V*	Isotype of heavy strain
2A	IGKV1-133*01	IGHV14-3*02	IgG1
2AB	IGKV5-43*01	IGHV3-8*02	IgG1
2HD	IGKV8-30*01	IGHV1-7*01	IgG1

### Sequence analysis of anti-Scl70 binders and implications for antigen binding

**Figure 4:**
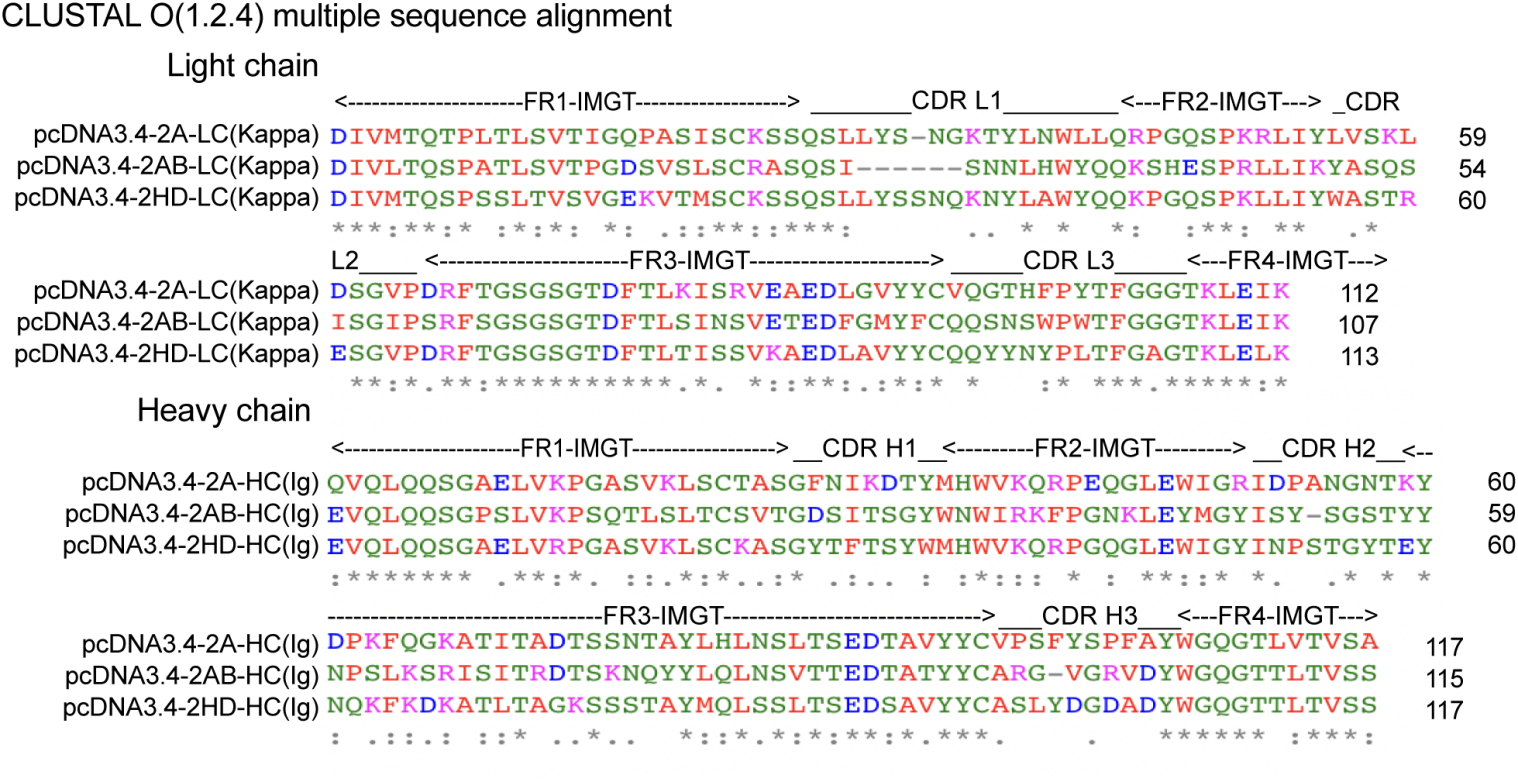
Alignment of the amino acid sequences of three scFv (2A, 2AB, and 2HD) identified. FRs and CDRs were defined by VBASE2. Positions of the respective CDRs for the variable domains of the heavy chain (CDR H1-3) and light chain (CDR L1-3) are indicated. FR1-4 represent the FRs of the antibody domains. The color of the amino acid represents their physicochemical properties: red, small (small+hydrophobic [including aromatic, Y]); blue, acidic; magenta, basic-H; green, hydroxyl+sulfhydryl+amine+G. [*] denote perfect alignment, fully conserved residue; [:] denote strongly similar conservation; and [.] denote weakly similar conservation.

To explore the molecular basis of the differences in the affinity of 2A, 2AB, and 2HD, their amino acid sequences were further assessed using Clustal Omega (1.2.4) (26) for aligning the sequences and the FR and CDR regions were identified by VBASE2 (27). Overall, the FR regions were found to be highly conserved, whereas the amino acid sequences shared relatively low sequence homology in the CDR area (**Figure 4**). The paratope, which is the antigen-binding site of the antibody and this case was composed of six CDR areas, was the molecular basis of the observed binding affinity differences.

**Figure 5:**
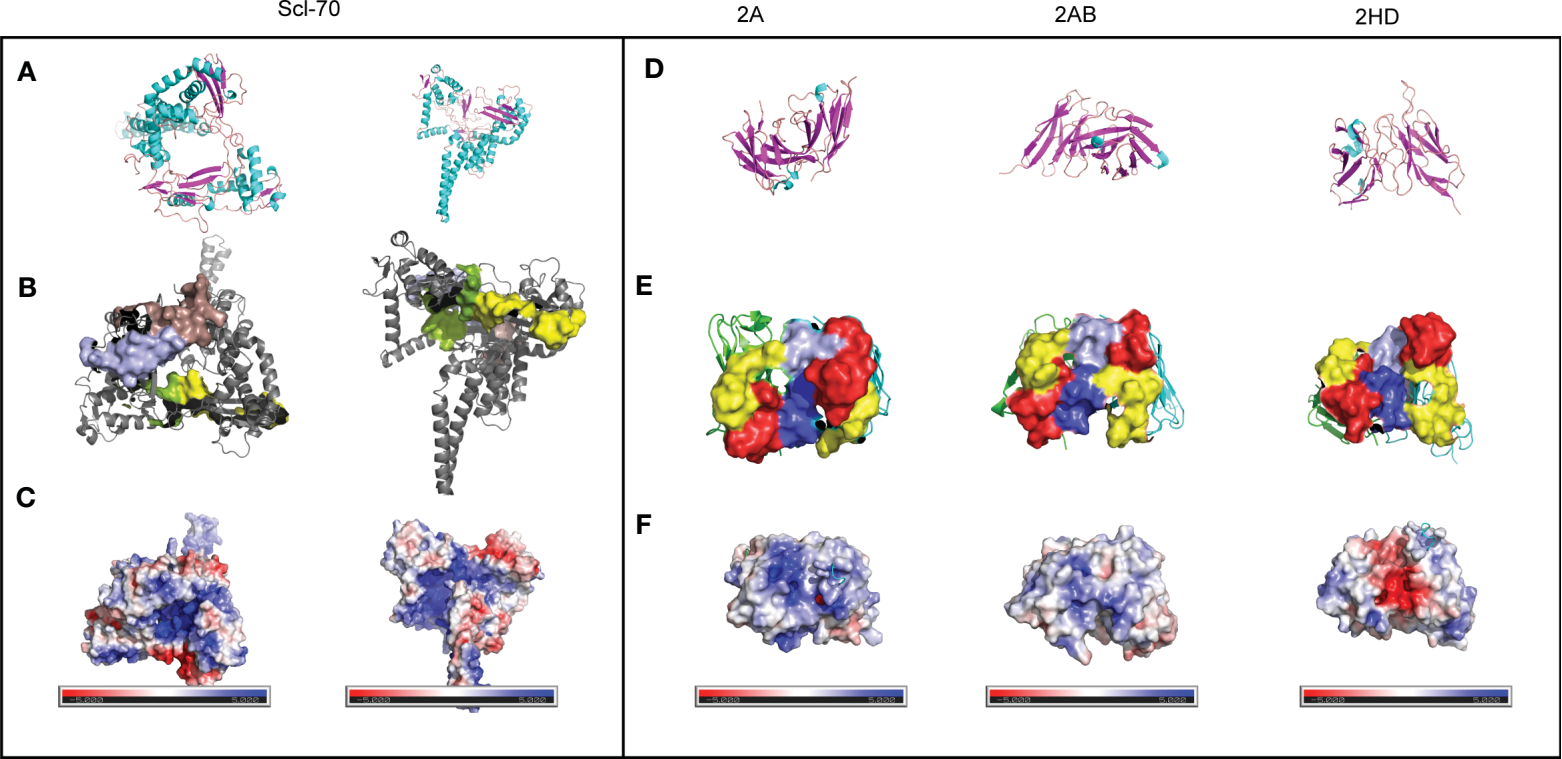
Preliminary modeling of the paratope of the three scFvs and epitope of the Scl-70 antigen. **(A)** Secondary structure of the Scl-70 antigen based on the published 3D structure 1r49. Helix: cyan; sheet: magenta; loops: orange. **(B)** 3D structure of two known antigen epitopes: tints (epitope 1) and light blue (epitope 3). 3D structure of the protein surface of two other known antigen epitopes: green (epitope 2) and yellow (epitope 4). **(C)** Electrostatic surface of epitope on the Scl-70 antigen, color coded from negative (red) to positive (blue) charge. **(D)** Secondary structure of scFv. Helix: cyan; sheet: magenta; loops: orange. **(E)** Protein surface of the paratope of the three scFv. The heavy chain is green, light chain is cyan, red surface is CDR1, yellow surface is CDR2, light blue surface is CDR3 from the light chain, and blue surface is CDR3 from heavy chain. **(F)** Electrostatic surface of the paratope on the antibody, color coded from negative (red) to positive (blue) charge.

The low sequence homology of the CDRs suggested that the relationship between the 3D structure of scFvs and the antigen should be investigated further. The sheets and helices were distributed in the FR regions, and the six CDR regions were mainly loops (**Figures 5A, D**. The known epitopes of the Scl-70 antigen have been reported as follows: epitope 1 (205–224, WWEEERYPEGIKWKFLEHKG), epitope 2 (349–368, RIANFKIEPPGLFRGRGNHP), epitope 3 (397–416, PGHKWKEVRHDNKVTWLVSW), and epitope 4 (517–536, ELDGQEYVVEFDFLGKDSIR) (20). Next, we annotated the known epitopes of the antigen (**Figure 5B**) and the antibody (**Figure 5E**). The electrostatic potential of the surface of the antigen (**Figure 5C**) and antibodies (**Figure 5F**) based on the APBS calculation were plotted alongside the potential color code. In the antigen, the epitope 2 (green) region was positively charged compared with the other regions. In the 2A antibody, the CDRs were predicted to be more positively charged than in the other two antibodies. In the 2HD antibody, CDRs were predicted to be highly negatively charged in the CDR3 region of the heavy chain. The CDRs of the 2AB antibody appeared to be balanced.

The electrostatic potential distribution in the CDR regions of 2A, 2AB, and 2HD varied from positive to negative (**Figure 5F**). The physicochemical properties of the amino acids determined the electrostatic potential, with KRH being the basic amino acids and BDENQZ the acidic amino acids. Regarding the differences in the amino acid composition (**Figure 4**), the percentages of basic amino acid in the CDRs were 7.35% (5/68), 4.92% (3/61), and 2.9% (2/69) in 2A, 2AB, and 2HD, respectively. The percentages of acidic amino acids in the CDRs were 16.18% (11/68), 18.03 % (11/61), and 21.74% (15/69) in 2A, 2AB, and 2HD, respectively. However, the theoretical isoelectric point of the scFv of 2A (8.58) was highest than 2AB (8.33) and 2HD (7.81). The overall amino acid percentage of the scFv fragments was determined to be 10.04% (23/229), 8.56% (19/222), and 8.7% (20/230) in 2A, 2AB, and 2HD, respectively. The percentages of acidic amino acids were 16.59% (38/229), 17.12 % (38/222), and 16.96% (39/230) in 2A, 2AB, and 2HD, respectively. These findings suggest differentially charged patches within the para- and epitopes may have yielded the electrostatic interaction needed for binding.

### Expression and purification of murine-human chimeric antibodies

In our previous work, an optimized pcDNA3.4 with the constant regions of the kappa chain, the heavy chain domain of *Homo sapiens*, and signal peptides was constructed. The variable regions of the kappa light chain and the heavy chains of 2A, 2AB, and 2HD were inserted into the optimized pcDNA3.4. The amino acid sequences of antibodies were verified (Material S1) before transfection.

CHO cells were transfected with the verified vectors, and the expression of the three antibodies was identified by SEC-HPLC 6 days post-transfection (**Table 2**). Compared with the other antibodies, 2HD exhibited the highest levels. The antibodies were purified by protein affinity chromatography and the purity was confirmed by size-exclusion gel chromatography (**Table 2**, **Figure S2A**). The non-reduced and reduced SDS-PAGE revealed the stability of the recombinant mAbs (**Table 2**, **Figure S2B**).

**Table 2:** Expression of the three antibodies identified by size-exclusion gel chromatography (SEC).

Clone ID	Expression(μg/mL)	Total after purification (mg)	SEC (%)	Scl-70 ELISA binding (EC_50_)^a^
2A	44.0	0.4	96.28	0.00431
2AB	64.8	0.7	99.45	0.02719
2HD	83.1	0.9	99.99	0.03683

a EC_50_, half maximal effective concentration.

### Analysis of the specificity and affinity of the murine-human chimeric antibodies

**Figure 6:**
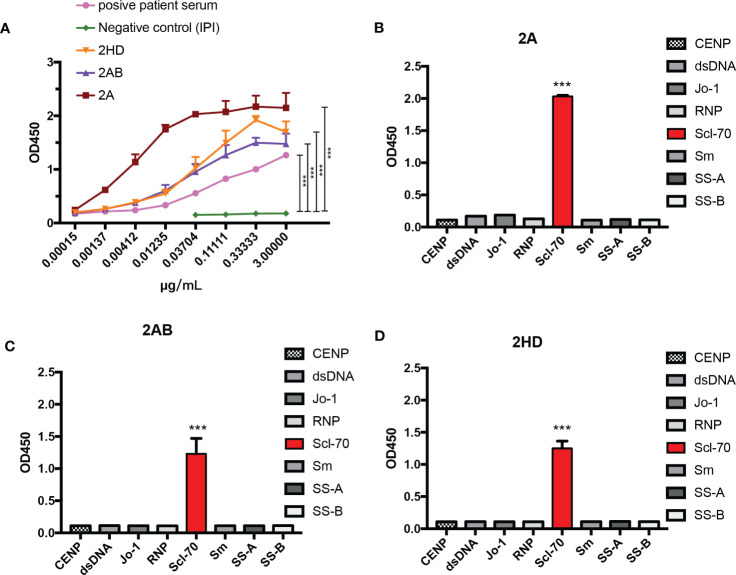
Analysis of the specificity and affinity of three chimeric Scl-70-specific humanized antibodies. **(A)** Affinity analysis by ELISA. Data represents the mean ± standard deviation of one experiment performed with triplicates. ***p < 0.001 by an ANOVA test. **(B–D)** Specificity analysis by ELISA of the chimeric antibody **(B)** 2A, **(C)** 2AB, and **(D)** 2HD. ***p < 0.001 by analysis of variance with Tukey’s *post-hoc* test.

To further test the affinity of our murine-human chimeric antibodies (2A, 2AB, and 2HD), an affinity ELISA was performed. As shown in **Figure 6A**, all the antibodies reacted with the antigen, as well as with the positive human serum, with all three chimeric antibodies exhibiting lower binding half maximal effective concentration (EC_50_) values (2A: 0.04311 μg/mL, 2AB: 0.02719 μg/mL, and 2HD: 0.03683 μg/mL) than that of the positive patient serum (0.08483 μg/mL) (**Table 2**).

Determination of the specificity of the three chimeric antibodies showed that all purified chimeric antibodies were specific to the Scl-70 antigen and no cross-binding was observed with other antinuclear antibody antigens (**Figures 6B–D**). These results indicated that the herein produced murine-human chimeric antibodies (2A, 2AB, and 2HD) have high affinity and specificity for Scl-70 and may be used as an antibody reagent to detect Scl-70 antigen or a standard reference replaced for positive patient serum.

## Discussion

Due to the unclear pathogenesis of SSc, some studies attributed it to an exacerbated immune response with production of cytokines that stimulate fibroblasts to be overactive, resulting in the deposition of a large amount of collagen and cell matrix in tissues, which will in turn damage the vascular endothelial cells and cause vascular stenosis and occlusion (4). However, in 1988, Douvas et al. found that compared to non-collagen genes, the core binding motif of topoisomerase I enriched in the exon-intron junctions of the fibrillar collagen gene and the promoter region of the dermal collagen gene alpha1 and alpha2 (28). Because overexpression of collagen and production of autoantibodies against Scl-70 were common in systemic sclerosis. And the main function of topoisomerase I is to reconstruct DNA to facilitate nuclear processes such as DNA replication (29). These hint overexpression of ATA related to a mechanism of excessive production of collagen and the research targeting Scl70/topoisomerase I may help developing an approach to treating the disease.

The ATA is a serum marker for SSc that is recognized as an indicator of poor prognosis, which is often closely related to diffuse skin lesions, proximal skin involvement, cardiac involvement, concurrent tumors, and pulmonary fibrosis (30). However, when preparing *in vitro* detection reagents to detect ATA, the use of antibodies derived from human sera or plasma containing known specific antibodies can be a challenge (6). Herein, a new strategy to prepare functional humanized anti-Scl-70 mAbs that can be used as substitute for patient serum is described.

Phage display technology has been successfully used for the discovery of hundreds of antibodies for research, diagnostics, and therapeutic applications. In M13 phage, the fusion protein is transferred to the periplasm in an unfolded state. In addition, fusion and binding of phage coat proteins with their fusion partners occurs in the periplasm (31). This property is advantageous for some proteins, including secretory heteroproteins, but detrimental to cytoplasmic proteins. T7, T4, and λ are released as lytic phages upon cell lysis and are fully assembled in the cytoplasm (31). In these phages, the displayed proteins can fold within the cytoplasm without entering the periplasmic region. Hence, lysing phages are better suited for displaying cytosolic and nuclear proteins than filamentous phages assembled in the periplasmic environment. Additionally, the use of lytic phages has aggravated practical work. Therefore, filamentous phages are more suitable than lytic phages for the synthesis of secretory proteins and antibodies (32). Hence, in this study, the M13 phage was selected based on the targeted secreted protein, anti-Scl-70. Furthermore, M13KO7, which contains an antibiotic (kanamycin) resistance gene and the P15A open reading frame, allowing its genome to be replicated as a plasmid in *Escherichia coli*, was used as the helper phage of the M13 phage.

In 2003, Weber et al. provided a modified version of the strategy to produce autoimmune antibodies requiring only a small amount of patient blood (8). Briefly, B cells were isolated and expanded *in vitro*, followed by the generation of the scFvs libraries. Finally, the four anti-Scl-70 scFvs were isolated by phage screening, which was twice as only two scFvs obtained by traditional phage screening using 10 times the patient’s blood directly (8). Our noval strategy is more efficient and biodiversity than Weber et al.’s method, obtaining 10 high-affinity scFvs without any human specimen. Finally, we humanized the antibody to be more convenient for clinical application, whether it is for diagnosis.

The affinity of the antibody was composed of electrostatic attraction, van der Waals forces, hydrogen bonds, and hydrophobic interactions, among which hydrophobic interactions were the strongest followed by electrostatic attraction. Although humanized macroregions may change the conformation of the Fab region of murine mAbs and affect their ability to bind antigens (33), our humanized antibodies showed similar affinity to the antigen. In this study, 2A showed the most affinity for Scl-70 than 2AB and 2HD. Because the known Scl-70 epitopes strongly vary in their IEP (1: 5.68, 2: 12.22, 3:10.50 and 4: 3.82), the epitope 1 and epitope 4 of Scl-70 seem higher probability binding to the three scFvs in this study. 2A exhibit the highest isoelectric point (IEP) in paratope that may explain 2A had a slightly higher affinity for Scl-70 than 2AB and 2HD. In contrast, the electrostatic potential of a protein can affects its expression due to overexpression-related toxicity (33). This may explain the differences observed concerning the levels of the three antibodies. However, the CDR region of 2AB was shorter than that of 2A and 2HD, and the electrostatic potential of the CDR surface was more balanced. Due to the negative potential on the surface of the cell, the CDR region of the antibody with high positive electrostatic potential was phagocytosed by cells more easily, resulting in easier degradation as compared with the other antibodies, while negative electrostatic potential reduced pinocytosis and elimination of the antibody. Phage display technology has been reported to introduce positive electrostatic potential on the surface of variable regions of antibodies under panning pressure, resulting in nonstandard Fc binding (34).

## Conclusion

We have shown a noval strategy of obtaining scFvs of high affinity to Scl-70, major autoantigen of Scc, for the first time. Without patient serum, this new method provides 2.5-fold higher yield than the improved method needing a little patient serum and 5-fold higher yield than the traditional method requiring large volume of patient serum. Three novel anti-Scl-70 chimeric antibodies were cloned, expressed, and characterized their ability for standard reagents in diagnostic immunoassays for SSc. Comparison between the 3D structure of the paratope of the scFvs and the epitopes against the Scl-70 revealed the differences between electrostatic surface of the paratope on the antibodies may affect the affinity of three scFvs. And the differences of distribution and total amount of acidic amino acids and basic amino acids determined the different IEPs which were the basis of electrostatic surface. The finding of 3 novel anti-Scl-70 antibodies with their preliminary modeling and sequences of scFvs will certainly provide new foundations for developing enhanced diagnostic strategies for SSc and worth to make available for the scientific community.

## Data availability statement

The sequences of the genes encoding the Variable region of three antibodies 2A, 2AB and 2HD were upload in GenBank under the accession numbers OP729704, OP729705 for 2A; OP729706, OP729707 for 2AB; OP729708, OP729709 for 2HD.

## Ethics statement

The studies involving human participants were reviewed and approved by the Ethics Committee of Fujian Provincial Hospital, Fuzhou, China (No. K2022-04-010). The patients/participants provided their written informed consent to participate in this study. The animal study was reviewed and approved by the Institutional Animal Care and Use Committee of Fudan University (No. 8177141042).

## Author contributions

SC and QH contributed to conception and design of the study. SC, QL and YZ performed all the experiments. QH construct the model. QH analysed all the data. QH wrote the first draft of the manuscript. SC wrote sections of the manuscript. All authors contributed to the article and approved the submitted version.
