# Supplementary material for: Human-murine chimeric autoantibodies with high affinity and specificity for systemic sclerosis
Source: Front Immunol. 2023 Jun 16;14:1127849. doi: 10.3389/fimmu.2023.1127849 (PMC10311643; doi:10.3389/fimmu.2023.1127849)
Supplement: Supplementary file 1 [file DataSheet_1.docx]

Supplementary Material

Evaluation and preparation of diagnostic chimeric anti-Scl-70 antibody for systemic sclerosis

Sunhui Chen^1,4^, Qiong Liang^1,4^, Yanhang Zhuo^1,2^, Qin Hong^1,3,4,*^

*** Correspondence:** Qin Hong: hqlkjx@163.com

# Supplementary Data

**Light chain：**

***pcDNA3.4-2A-LC(Kappa)***

DIVMTQTPLTLSVTIGQPASISCKSSQSLLYSNGKTYLNWLLQRPGQSPKRLIYLVSKLDSGVPDRFTGSGSGTDFTLKISRVEAEDLGVYYCVQGTHFPYTFGGGTKLEIKRTVAAPSVFIFPPSDEQLKSGTASVVCLLNNFYPREAKVQWKVDNALQSGNSQESVTEQDSKDSTYSLSSTLTLSKADYEKHKVYACEVTHQGLSSPVTKSFNRGEC

***pcDNA3.4-2AB-LC(Kappa)***

DIVLTQSPATLSVTPGDSVSLSCRASQSISNNLHWYQQKSHESPRLLIKYASQSISGIPSRFSGSGSGTDFTLSINSVETEDFGMYFCQQSNSWPWTFGGGTKLEIKRTVAAPSVFIFPPSDEQLKSGTASVVCLLNNFYPREAKVQWKVDNALQSGNSQESVTEQDSKDSTYSLSSTLTLSKADYEKHKVYACEVTHQGLSSPVTKSFNRGEC

***pcDNA3.4-2HD-LC(Kappa)***

DIVMTQSPSSLTVSVGEKVTMSCKSSQSLLYSSNQKNYLAWYQQKPGQSPKLLIYWASTRESGVPDRFTGSGSGTDFTLTISSVKAEDLAVYYCQQYYNYPLTFGAGTKLELKRTVAAPSVFIFPPSDEQLKSGTASVVCLLNNFYPREAKVQWKVDNALQSGNSQESVTEQDSKDSTYSLSSTLTLSKADYEKHKVYACEVTHQGLSSPVTKSFNRGEC

**Heavy chain：**

***pcDNA3.4-2A-HC(IgG1)***

QVQLQQSGAELVKPGASVKLSCTASGFNIKDTYMHWVKQRPEQGLEWIGRIDPANGNTKYDPKFQGKATITADTSSNTAYLHLNSLTSEDTAVYYCVPSFYSPFAYWGQGTLVTVSAASTKGPSVFPLAPSSKSTSGGTAALGCLVKDYFPEPVTVSWNSGALTSGVHTFPAVLQSSGLYSLSSVVTVPSSSLGTQTYICNVNHKPSNTKVDKKVEPKSCDKTHTCPPCPAPELLGGPSVFLFPPKPKDTLMISRTPEVTCVVVDVSHEDPEVKFNWYVDGVEVHNAKTKPREEQYNSTYRVVSVLTVLHQDWLNGKEYKCKVSNKALPAPIEKTISKAKGQPREPQVYTLPPSRDELTKNQVSLTCLVKGFYPSDIAVEWESNGQPENNYKTTPPVLDSDGSFFLYSKLTVDKSRWQQGNVFSCSVMHEALHNHYTQKSLSLSPGK

***pcDNA3.4-2AB-HC(IgG1)***

EVQLQQSGPSLVKPSQTLSLTCSVTGDSITSGYWNWIRKFPGNKLEYMGYISYSGSTYYNPSLKSRISITRDTSKNQYYLQLNSVTTEDTATYYCARGVGRVDYWGQGTTLTVSSASTKGPSVFPLAPSSKSTSGGTAALGCLVKDYFPEPVTVSWNSGALTSGVHTFPAVLQSSGLYSLSSVVTVPSSSLGTQTYICNVNHKPSNTKVDKKVEPKSCDKTHTCPPCPAPELLGGPSVFLFPPKPKDTLMISRTPEVTCVVVDVSHEDPEVKFNWYVDGVEVHNAKTKPREEQYNSTYRVVSVLTVLHQDWLNGKEYKCKVSNKALPAPIEKTISKAKGQPREPQVYTLPPSRDELTKNQVSLTCLVKGFYPSDIAVEWESNGQPENNYKTTPPVLDSDGSFFLYSKLTVDKSRWQQGNVFSCSVMHEALHNHYTQKSLSLSPGK

***pcDNA3.4-2HD-HC(IgG1)***

EVQLQQSGAELVRPGASVKLSCKASGYTFTSYWMHWVKQRPGQGLEWIGYINPSTGYTEYNQKFKDKATLTAGKSSSTAYMQLSSLTSEDSAVYYCASLYDGDADYWGQGTTLTVSSASTKGPSVFPLAPSSKSTSGGTAALGCLVKDYFPEPVTVSWNSGALTSGVHTFPAVLQSSGLYSLSSVVTVPSSSLGTQTYICNVNHKPSNTKVDKKVEPKSCDKTHTCPPCPAPELLGGPSVFLFPPKPKDTLMISRTPEVTCVVVDVSHEDPEVKFNWYVDGVEVHNAKTKPREEQYNSTYRVVSVLTVLHQDWLNGKEYKCKVSNKALPAPIEKTISKAKGQPREPQVYTLPPSRDELTKNQVSLTCLVKGFYPSDIAVEWESNGQPENNYKTTPPVLDSDGSFFLYSKLTVDKSRWQQGNVFSCSVMHEALHNHYTQKSLSLSPGK

**>OP729704 [organism=synthetic construct] Synthetic construct clone 2AB from Mus musculus, Variable region of humanized anti-Scl 70 immunoglobulin IgG1 heavy chain, partial cds**

GAGGTGCAGCTGCAGCAGTCAGGACCTAGCCTCGTGAAACCTTCTCAGACTCTGTCCCTCACCTGTTCTGTCACTGGCGACTCCATCACCAGTGGTTACTGGAACTGGATCCGGAAATTCCCAGGAAATAAACTTGAGTACATGGGGTACATAAGCTACAGTGGTAGCACTTACTACAATCCATCTCTCAAAAGTCGAATCTCCATCACTCGAGACACATCCAAGAACCAGTACTACCTGCAGTTGAATTCTGTGACTACTGAGGACACAGCCACATATTACTGTGCAAGAGGGGTGGGACGTGTTGACTACTGGGGCCAAGGCACCACTCTCACAGTCTCCTCA

**>OP729705 [organism=synthetic construct] Mus musculus scFv fragment of Synthetic construct clone 2AB humanized anti-Scl 70 immunoglobulin kappa light chain gene, partial cds**

GACATTGTGCTAACTCAGTCTCCAGCCACCCTGTCTGTGACTCCAGGAGATAGCGTCAGTCTTTCCTGCAGGGCCAGCCAAAGTATTAGCAACAACCTACACTGGTATCAACAAAAATCACATGAGTCTCCAAGGCTTCTCATCAAGTATGCTTCCCAGTCCATCTCTGGGATCCCCTCCAGGTTCAGTGGCAGTGGATCAGGGACAGATTTCACTCTCAGTATCAACAGTGTGGAGACTGAAGATTTTGGAATGTATTTCTGTCAACAGAGTAACAGCTGGCCGTGGACGTTCGGTGGAGGCACCAAGCTGGAAATCAAA

**>OP729706 [organism=synthetic construct] Synthetic construct clone 2A from Mus musculus, Variable region of humanized anti-Scl 70 immunoglobulin IgG1 heavy chain, partial cds**

CAGGTTCAGCTGCAGCAGTCTGGGGCAGAGCTTGTGAAGCCAGGGGCCTCAGTCAAGTTGTCCTGCACAGCTTCTGGCTTCAACATTAAAGACACCTATATGCACTGGGTGAAGCAGAGGCCTGAACAGGGCCTGGAGTGGATTGGAAGGATTGATCCTGCGAATGGTAATACTAAATATGACCCGAAGTTCCAGGGCAAGGCCACTATAACAGCTGACACATCCTCCAACACAGCCTACCTGCACCTCAACAGCCTGACATCTGAGGACACTGCCGTCTATTACTGTGTTCCCTCTTTCTACTCCCCGTTTGCTTACTGGGGCCAAGGGACTCTGGTCACTGTCTCTGCA

**>OP729707 [organism=synthetic construct] Mus musculus scFv fragment of Synthetic construct clone 2A humanized anti-Scl 70 immunoglobulin kappa light chain gene, partial cds**

GACATTGTGATGACCCAAACTCCACTCACTTTGTCGGTTACCATTGGACAACCAGCCTCTATCTCTTGCAAGTCAAGTCAGAGCCTCTTATATAGTAATGGAAAAACCTATTTGAATTGGTTATTACAGAGGCCAGGCCAGTCTCCAAAGCGCCTAATCTATCTGGTGTCTAAACTGGACTCTGGAGTCCCTGACAGGTTCACTGGCAGTGGATCAGGAACAGATTTTACACTGAAAATCAGCAGAGTGGAGGCTGAGGATTTGGGAGTTTATTACTGCGTGCAAGGTACACATTTTCCGTACACGTTCGGAGGGGGGACCAAGCTGGAAATAAAA

**>OP729708 [organism=synthetic construct] Synthetic construct clone 2HD from Mus musculus, Variable region of humanized anti-Scl 70 immunoglobulin IgG1 heavy chain, partial cds**

GAGGTCCAGCTGCAGCAGTCAGGGGCTGAGCTGGTGAGGCCTGGGGCTTCAGTGAAGCTGTCCTGCAAGGCTTCTGGCTACACCTTTACTAGCTACTGGATGCACTGGGTAAAACAGAGGCCTGGACAGGGTCTGGAATGGATTGGATACATTAATCCTAGCACTGGTTATACTGAGTACAATCAGAAGTTCAAGGACAAGGCCACATTGACTGCAGGCAAATCCTCCAGCACAGCCTACATGCAACTGAGCAGCCTGACATCTGAGGACTCTGCAGTCTATTACTGTGCAAGCCTCTATGATGGTGACGCCGACTACTGGGGCCAAGGCACCACTCTCACAGTCTCCTCA

**>OP729709 [organism=synthetic construct] Mus musculus scFv fragment of Synthetic construct clone 2HD humanized anti-Scl 70 immunoglobulin kappa light chain gene, partial cds**

GACATTGTGATGACACAGTCTCCATCCTCCCTAACTGTGTCAGTTGGAGAGAAGGTTACTATGAGCTGCAAGTCCAGTCAGAGCCTTTTATATAGTAGCAATCAAAAGAACTACTTGGCCTGGTACCAGCAGAAACCAGGGCAGTCTCCTAAACTGCTGATTTACTGGGCATCCACTAGGGAATCTGGGGTCCCTGATCGCTTCACAGGCAGTGGATCTGGGACAGATTTCACTCTCACCATCAGCAGTGTGAAGGCTGAAGACCTGGCAGTTTATTACTGTCAGCAATATTATAACTATCCGCTCACGTTCGGTGCTGGGACCAAGCTGGAGCTGAAA

# Supplementary Figures and Tables

## Supplementary Figures


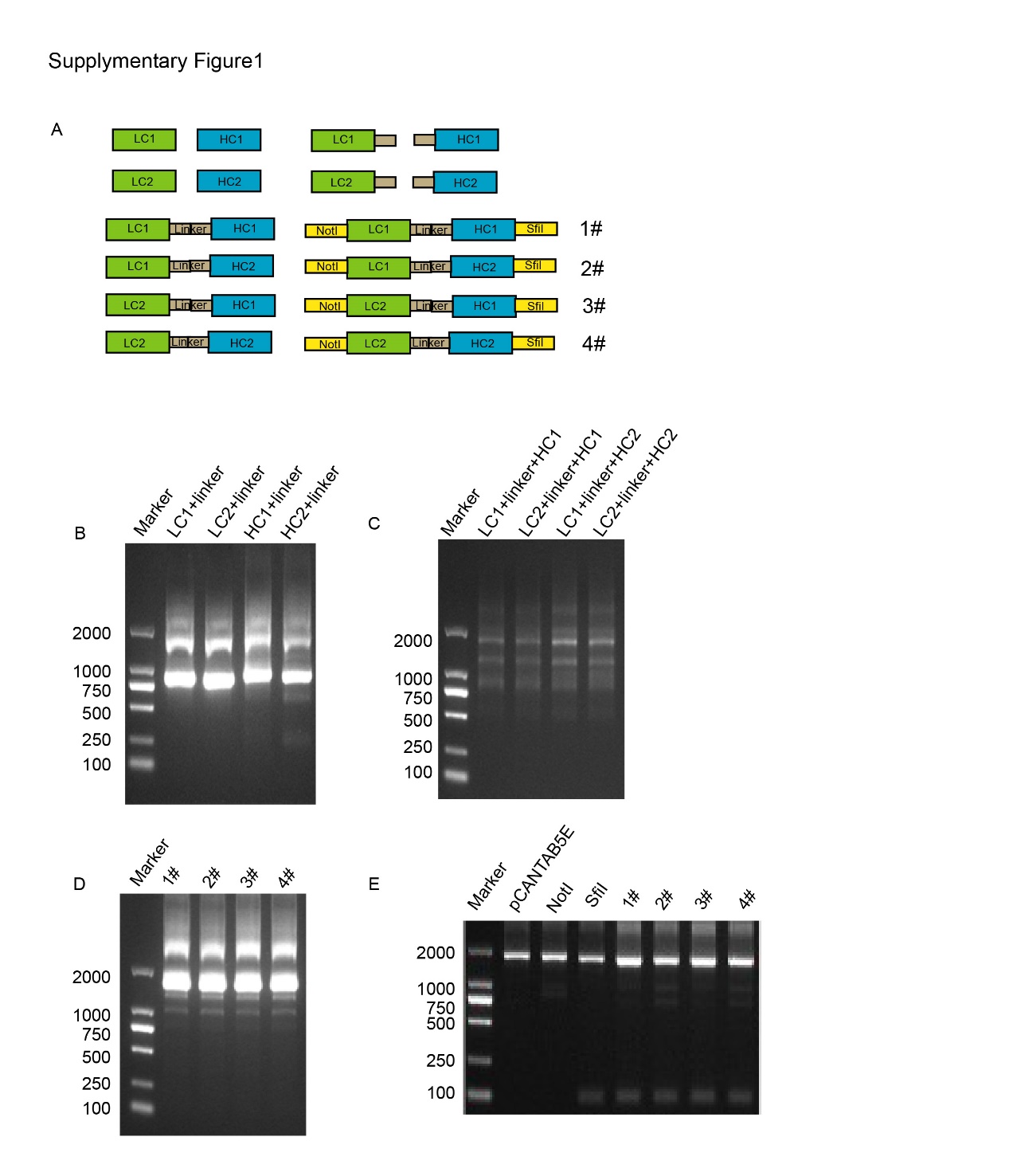


**Supplementary Figure 1.** Construction of recombinant library plasmid for mice ScFv. (A) Modular construced scheme of recombinant ScFv library from mice.


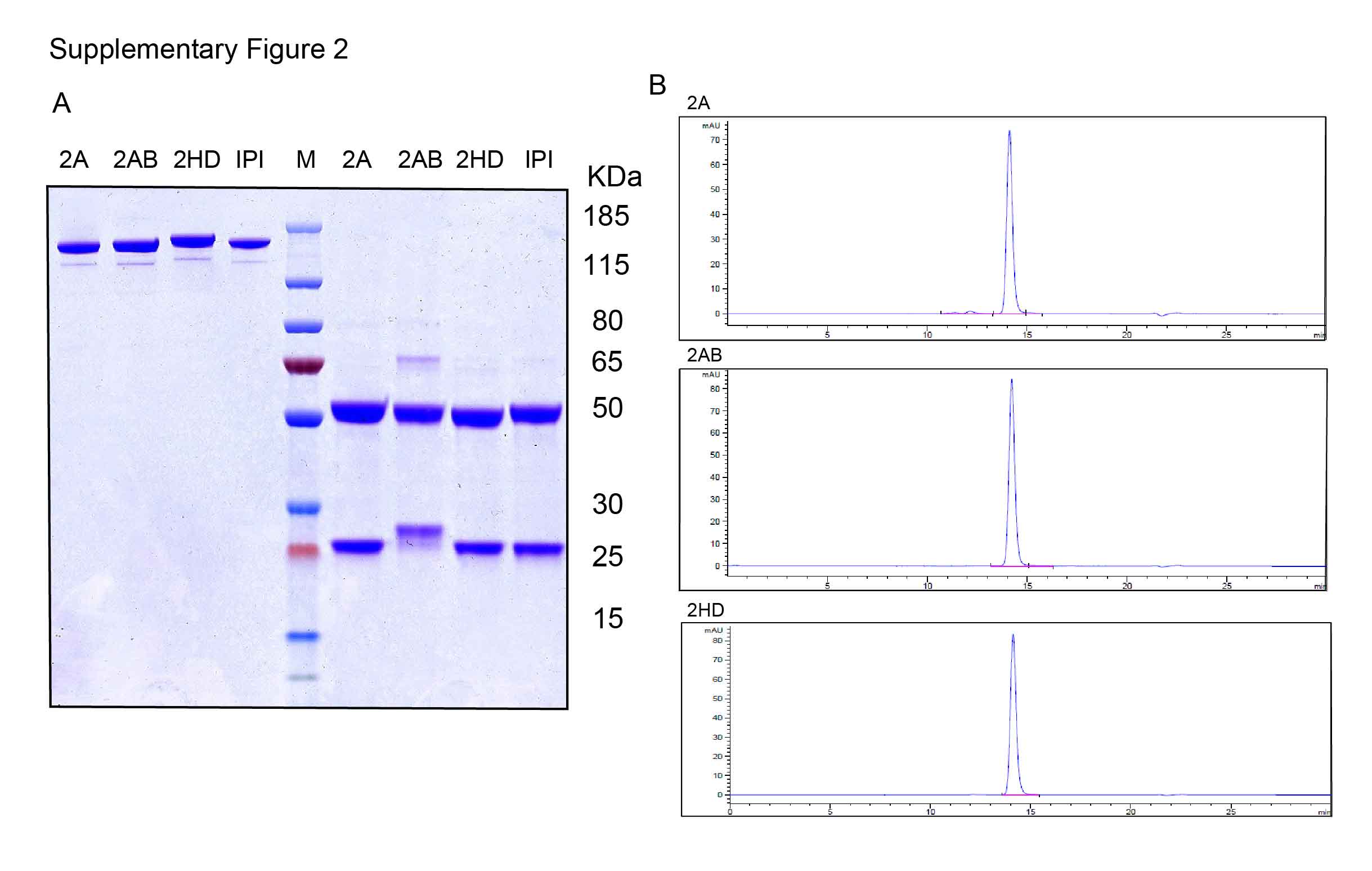


**Supplementary Figure 2** : Stability and Purity of chimeric antibodies 2A, 2AB and 2HD were identified by SDS-PAGE(A) and SEC(B).
